# Discovery of a new function of curcumin which enhances its anticancer therapeutic potency

**DOI:** 10.1038/srep30962

**Published:** 2016-08-01

**Authors:** Koji Nagahama, Tomoya Utsumi, Takayuki Kumano, Saeko Maekawa, Naho Oyama, Junji Kawakami

**Affiliations:** 1 Department of Nanobiochemistry, Frontiers of Innovative Research in Science and Technology, Konan University, 7-1-20 Minatojima-Minamimachi, Kobe 650-0047, Japan

## Abstract

Curcumin has received immense attention over the past decades because of its diverse biological activities and recognized as a promising drug candidate in a large number of diseases. However, its clinical application has been hindered due to extremely low aqueous solubility, chemical stability, and cellular uptake. In this study, we discovered quite a new function of curcumin, *i.e.* pH-responsive endosomal disrupting activity, derived from curcumin’s self-assembly. We selected anticancer activity as an example of biological activities of curcumin, and investigated the contribution of pH-responsive property to its anticancer activity. As a result, we demonstrated that the pH-responsive property significantly enhances the anticancer activity of curcumin. Furthermore, we demonstrated a utility of the pH-responsive property of curcumin as delivery nanocarriers for doxorubicin toward combination cancer therapy. These results clearly indicate that the smart curcumin assemblies act as promising nanoplatform for development of curcumin-based therapeutics.

Curcumin (CCM), a naturally-occurring polyphenol derived from the turmeric plant, has been received immense attention over the past decades because of its diverse biological activities, including anticancer, antioxidant, anti-amyloid, anti-inflammatory, antidiabetic, antibiotic, and antiviral activities^1^,^2^,^3^,^4^,^5^,^6^. Hence, CCM is recognized as a promising drug candidate in a large number of diseases such as cancer, neurodegenerative diseases, infectious diseases, and diabetes. However, the application of CCM in the therapeutic treatment has been hindered due to three obstacles. The first obstacle is extremely low aqueous solubility of CCM. CCM is hydrophobic molecule, and thus the maximum water solubility is about 30 nM, whereas the required concentration to exhibit various bioactivities is micro molar. Therefore, it is necessary to dissolve CCM in appropriate organic solvent for the use^7^. The second obstacle is chemical instability in aqueous condition. CCM quickly hydrolyze under physiological pH 7.4 in phosphate buffer with a half-life (*t*_1/2_) of only 20 min^8^,^9^. The third obstacle is low cellular uptake. It is demonstrated that CCM tends to deeply insert into the cell membrane through hydrophobic interaction with fatty acyl chains of lipids and hydrogen bonding with the phosphate group in a manner similar to cholesterol^10^,^11^, and thus limited amounts of CCM diffuse into the cytoplasm, while the main target action site of CCM for the most of its bioactivities is the cytoplasm. Therefore, overcoming these obstacles are the key challenges to achieve clinical application of CCM.

To date, several types of approaches have been performed to overcome these obstacles. For example, various kinds of chemical modification to CCM including polymer-conjugation have been performed^12^,^13^,^14^,^15^,^16^,^17^. Moreover, various types of nanomaterials including liposomes, polymeric micelles, and silica nanoparticles have been used as carrier materials for CCM^18^,^19^,^20^,^21^,^22^. In this context, we have adopted a supramolecular self-assembly approach to overcome these obstacles. We have recently reported the synthesis of amphiphilic CCM with several types of molecular architectures through conjugation of CCM with ethylene glycol oligomers and the fabrication of CCM nanovesicles (curcumisome) through supramolecular self-assembly of the CCM amphiphiles via hydrogen-bonding, π-π stacking, and hydrophobic interactions generated among the CCM segments (Fig. 1a). The CCM nanoassemblies exhibited several hundreds of thousands-fold higher aqueous dispersibility than CCM and significantly higher resistance against hydrolysis (*t*_1/2_: 168 h) than CCM, allowing intravascular administration of CCM at a high dose with keeping CCM’s structure intact for several days^23^.

Most recently, we serendipitously discovered a new function of the CCM nanoassemblies, *i.e.* pH-responsive endosomal disruption activity, when we examined cellular uptakes of these CCM nanostructures. In this study, we therefore selected anticancer activity as an example of biological activities of CCM, and investigated the impact of the pH-responsive endosomal disruption activity of CCM on its anticancer activity both *in vitro* and *in vivo*. Moreover, we investigated the molecular mechanism of the pH-responsive properties.

## Results

### Synthesis and characterization of CCM nanoassemblies

**Figure 1:**
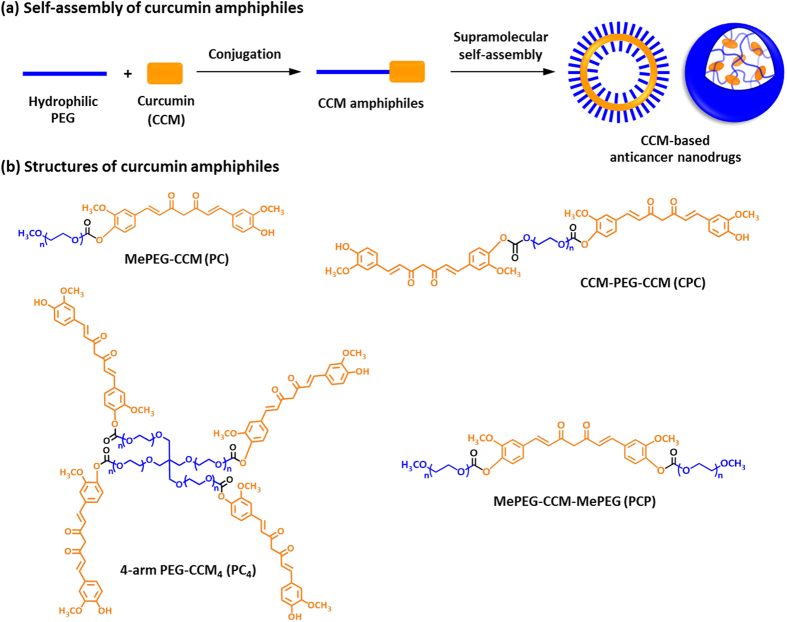
Schematic illustration of the synthesized CCM amphiphiles. (**a**) Formation of supramolecular nanoassemblies in aqueous conditions. (**b**) Structures of CCM amphiphiles synthesized in this study.

CCM amphiphiles with different numbers of CCM per molecule and hydrophilic/hydrophobic balance, PEG-CCM (PC), PEG-CCM-PEG (PCP), CCM-PEG-CCM (CPC), were synthesized by the same methods with our previous reports (Supplementary Fig. 1)^23^. In this study, we newly synthesized a type of CCM amphiphile with branched architecture, 4-arm PEG-CCM4 (PC_4_) (Fig. 1b). The characterizations of the CCM amphiphiles estimated by ^1^H-NMR (Supplementary Figs 2–5) and GPC analyses were summarized in Supplementary Table 1. Aqueous dispersions of the CCM amphiphiles were analyzed by DLS and TEM analyses (Supplementary Fig. 6). These dispersions showed a unimodal peak, and the averaged diameter was 178 ± 27 nm for PC, 195 ± 21 nm for PCP, and 205 ± 36 nm for CPC, and 79 ± 17 nm for PC_4_, respectively. PC, PCP and CPC nanoassemblies showed spherical shape, while PC_4_ nanoassemblies was square-shaped nanoparticles with multiple small CCM domains observed as dark spots in the TEM image. To investigate the architecture of nanoassemblies in molecular level, their ^1^H-NMR spectra were measured in D_2_O. As reported in our previous report^23^, the characteristic peaks of CCM segment (5.8–7.6 ppm) and the methylene peaks in PEG segment (3.7 ppm) were clearly observed in CDCl_3_ which is good solvent for CCM and PEG segments, while these peaks were completely disappeared in D_2_O and the methylene proton signal of PEG segment was detected as a sharp peak (Supplementary Figs 2–5). These results indicate that hydrophobic domains of CCM locates inside of the nanoassemblies and PEG segments extend out into aqueous environment at the surface of nanoassembly, as illustrated in Fig. 1a.

The critical assembly concentration (CAC) values of PC, PCP, CPC, and PC_4_, estimated from the eosin Y assay^24^, was 1.14 ± 10^−6^ M, 2.60 ± 10^−6^ M, 4.80 ± 10^−7^ M, and 3.54 ± 10^−6^ M, respectively. As reported in our previous report^23^, CCM amphiphiles with higher CCM contents were prone to show lower CAC, indicating that main driving force to form nanoassemblies is a couple of interactions between CCM segments. These interactions were characterized by FTIR analyses to be intermolecular π-π stacking among aromatic groups and π-conjugated linker in CCM segments, hydrogen bonding among ketone groups in CCM segments, and hydrophobic interaction of CCM segments.

### Cellular uptakes of CCM nanoassemblies

**Figure 2:**
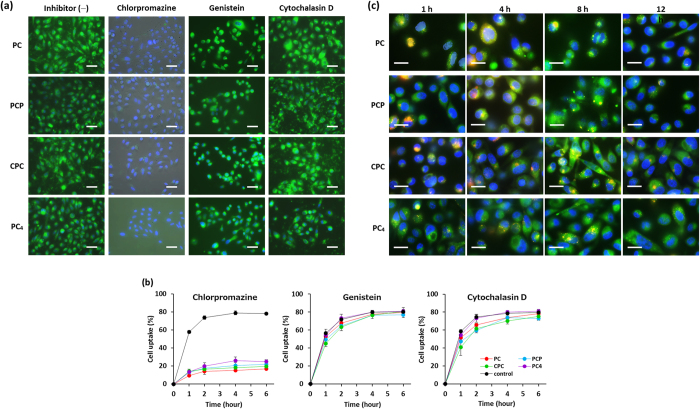
(**a**) Fluorescence microscope images of PC-3 cells treated with CCM nanoassemblies in the presence of endocytosis inhibitors. Green: CCM nanoassemblies, blue: nuclei. Scale bars indicate 50 μm. (**b**) Cell uptake of CCM nanoassemblies by PC-3 cells in the presence of endocytosis inhibitors. Values are average of three separate experiments in triplicate and are expressed as mean ± SD. (**c**) Fluorescence microscope images of PC-3 cells treated with CCM nanoassemblies. Green: CCM nanoassemblies, blue: nuclei, red: late endosomes/lysosomes. Scale bars indicate 20 μm.

Efficient cytoplasmic delivery of CCM is crucial challenge to enhance the biological activity. We investigated cellular uptakes of CCM nanoassemblies by human cancer cells (PC-3). CCM exhibits green fluorescence in aqueous condition, allowing the monitoring of cellular uptake and intracellular distribution of CCM-based nanoassemblies by fluorescence microscopy. Figure 2a (inhibitor −) shows CLSM images of PC-3 cells treated with four types of CCM nanoassemblies at equivalent CCM concentration. As a negative control sample, cellular uptake of free CCM was also examined. Although very weak green fluorescence was detected in PC-3 cells after 6 h incubation with free CCM (Supplementary Fig. 7), much strong fluorescence was observed in cells treated with CCM-based nanoassemblies.

To characterize the pathway of cell uptakes, endocytosis inhibitors were used to perturb entry. In this study, inhibitors which affect endocytic pathways in a more general manner were used. In particular, clathrin-mediated endocytosis, caveolae/raft-mediated endocytosis, and micropinocytosis were inhibited by chlorpromazine, genistein, and cytochalasin D, respectively. As shown in Fig. 2, cellular uptakes of all types CCM nanoassemblies was drastically reduced by chlorpromazine, while the cellular uptakes was also slightly reduced by cytochalasin D. This result indicates that the CCM nanoassemblies were mainly entered into cancer cell through clathrin-mediated endocytic pathway. Clathrin-mediated endocytosis is the most understood pathway. In this pathway, nanoparticles were transport into cells within vesicles, referred as early endosomes, and the endosomes either were recycled and exocytose out of the cell or maturated within cells. During endosome maturation process, early endosomes are fused with cytoplasmic vesicles and matured into late endosomes and lysosomes^25^. The entrapment of CCM nanoassemblies within endosomes is undesirable because CCM is rapidly degraded in the matured endosomes/lysosomes. Therefore, intracellular fate of CCM nanoassemblies after clathrin-mediated endocytosis was investigated with focusing on endolysosomal network. Endosomes and lysosomes were stained with Lysotracker Red giving a red color, and the nuclei were counterstained with Hoechst33342 giving blue color. Figure 2c shows CLSM overlap images of PC-3 cells treated with CCM nanoassemblies at equivalent CCM concentration. All types of CCM nanoassemblies rapidly entered into the cancer cells and yellow fluorescent spots were observed at early stage (1 h), indicating colocalization of nanoassemblies with endosomes. Importantly, green fluorescence arising from CCM nanoassemblies was also observed in cytoplasm even after early stage, indicating cytoplasmic distribution of nanoassemblies. An obvious increase in the green fluorescence intensity in cytoplasm was also seen over time for all types of nanoassemblies. Moreover, the decrease in the numbers of red and yellow spots was clearly observed over time. These results suggest that the maturation process of early endosomes to late endosomes and lysosomes is interrupted in the presence of CCM nanoassemblies. Additionally, the amount of nanoassemblies in cytoplasm clearly increased over time, indicating release of CCM nanoassemblies from endosomes to cytoplasm.

### pH-responsive properties of CCM nanoassemblies

**Figure 3:**
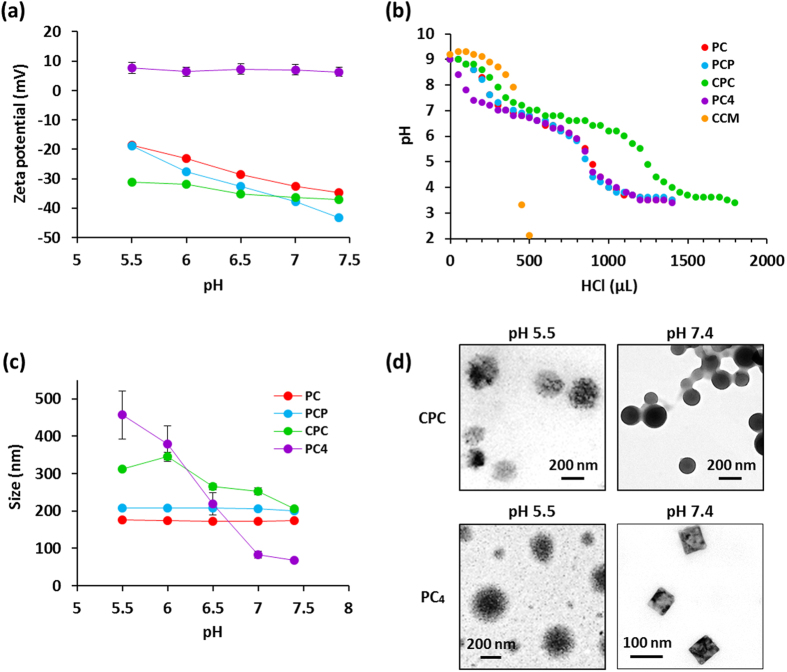
(**a**) Influences of pH on the zeta-potential of CCM nanoassemblies. (**b**) Acid-base titration profiles of CCM nanoassemblies in water at 37 °C. (**c**) Influences of pH on the size of CCM nanoassemblies. (**d**) TEM images of CCM nanoassemblies (CPC and PC_4_) prepared at pH 7.4 and pH 5.5, respectively. Values are average of three separate experiments in triplicate and are expressed as mean ± SD.

The maturation of endocytic vesicles into late endosome and lysosome is characterized by acidification from pH 7.4 to 4.5 within the vesicle^26^. Considering the rapid cytosolic dispersion of CCM nanoassemblies, we hypothesized that the CCM nanoassemblies would have pH-responsive property under endosomal acidic pH condition, and the pH-responsive property could contributes to the cytosolic dispersion. Therefore, we investigated pH-responsive properties of CCM nanoassemblies. We first examined the effect of pH decrease on the zeta potential of nanoassemblies. The average zeta potential of nanoassemblies at different pH values are shown in Fig. 3a. At pH 7.4, PC, PCP, and CPC nanoassemblies had a negative zeta potential ranging from −35 to −43 mV, while PC_4_ nanoassembly had a positive zeta potential (+5 mV). When pH values decreased from 7.4 to 5.5, the gradual increase in zeta potential was observed for all types of nanoassemblies. In particular, PC and PCP nanoassemblies showed more increase in the zeta potential than CPC and PC_4_ nanoassemblies. These results suggest that the CCM nanoassemblies have an ability to adsorb H^+^ ions. Considering the significant increase in zeta potential for PC and PCP and the slight increase for CPC and PC_4_ nanoassemblies, it is suggested that PC and PCP nanoassemblies adsorb H^+^ ions at the near surface, while CPC and PC_4_ nanoassemblies adsorb H^+^ ions inside of nanoassemblies.

**Figure 4:**
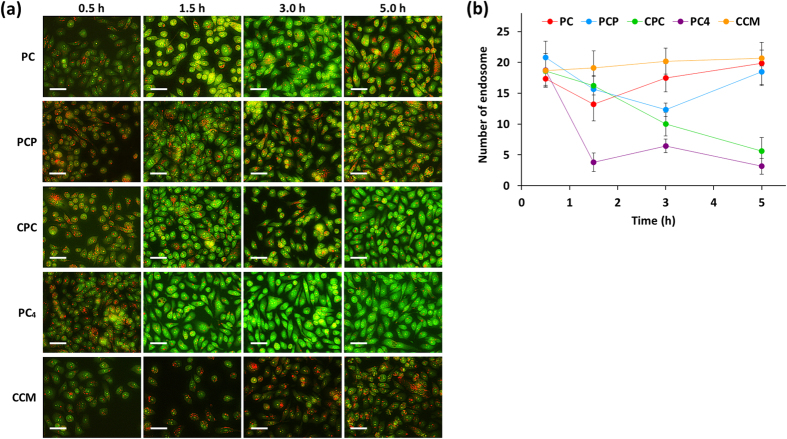
(**a**) Fluorescence microscopy images of PC-3 cells treated with CCM nanoassemblies in the presence of acridine orange. Green: CCM nanoassemblies, red: late endosome/lysosome. Scale bars indicate 50 μm. (**b**) Time course of the number of endosomes/lysosomes in PC-3 cells treated with CCM nanoassemblies. Values are average of three separate experiments in triplicate and are expressed as mean ± SD.

We next examined pH buffering capacity of CCM nanoassemblies derived from the H^+^ ion adsorption. The acid-base titration curves of the CCM nanoassemblies are shown in Fig. 4b. It is noteworthy that, all types of CCM nanoassemblies had an obvious pH buffering capacity between pH 7.2 to 5.6, corresponding to pH value within endosomes during the maturation. The buffering capacity of PC and PCP was almost same. The buffering capacity of CPC and PC_4_ nanoassemblies were 1.5-fold and 1.4-fold higher than PC and PCP nanoassemblies, respectively. The p*K*_a_ values were estimated to be pH 6.4 for all types of nanoassemblies, indicating that the origin of buffering capacity is the same. It is well known that PEG does not have pH buffering capacity because nonionic PEG can not act as proton acceptor^27^. Considering the report, the proton acceptor of CCM nanoassemblies can be assigned to the CCM segments. CCM displays typical keto-enol tautomerism as illustrated in Supplementary Fig. 8. CCM has three proton acceptable sites per a molecule, two phenolic hydroxyl groups and a diketone group (keto form) or an enolic hydroxyl group (enol form)^3^. PCP molecule, which both two phenolic hydroxyl groups were used to the conjugation with PEG, also showed buffering capacity and the capacity was similar with PC having a free phenolic hydroxyl group. Considering this, it is plausible that the diketone group or enolic hydroxyl group of CCM segments is the possible proton acceptor. In contrast, free CCM in aqueous solution with equivalent concentration at CCM level did not show any buffering capacity (Fig. 3b). These results suggest that the buffering capacity is attributed to the nanoassembly formations of CCM. To assess the thought, we examined pH buffering capacity of CCM-PEG conjugates in water/methanol (70/30, v/v) mixed solution in which CCM-PEG conjugates do not form nanoassemblies. Supplementary Fig. 9 shows the acid-base titration curves of the CCM-PEG conjugates with equivalent concentration at CCM level. As we expected, CCM-PEG conjugates did not show any pH buffering capacity and the profiles were as same with free CCM. Importantly, it is thus concluded that the nanoassembly formations of CCM is an essential for exhibition of the pH buffering capacity.

The equilibrium between keto form and enol form of CCM is dependent on the molecular environment, including pH in the solution and its states of matter (solid or liquid)^28^. As described above, the diketone group and enolic hydroxyl group of the CCM segments in nanoassemblies is potential proton acceptor related to the pH buffering capacity. It was reported that the keto form dominates at acidic or neutral pH, while the enol form is favored at alkaline pH. The p*K*_a_ value of enol group and two phenolic hydroxyl groups in aqueous solution is 8.38, 9.88, and 10.51, respectively^29^. The facts indicate that most of enolic hydroxyl groups is protonated at neutral pH and thus cannot act as proton acceptor at acidic pH. On the other hand, Akulov and co-workers have reported that diketo oxygen atoms in CCM adsorb a H^+^ ion in weak acidic condition and the protonated forms are a stable six-membered ring as shown in Supplementary Fig. 8 30. Moreover, Basnet and co-workers have reported that the keto form dominates in solid state, and the keto form acts as a potent proton acceptor in the solid state^31^. Consequently, the proton acceptor for the pH buffering capacity of CCM-based nanoassemblies can be assigned to diketone group of the CCM segments. Thus, it was demonstrated that the formation of supramolecular nanoassembly allows CCM molecules to maintain keto form through the formation of solid state CCM domains in nanoassemblies, and the pH buffering capacity was successfully achieved. This is the first discovery on pH buffering capacity of CCM. Based on the above results, the CCM nanoassemblies probably exhibit the “proton sponge effect” in endosomes/lysosomes.

Then, we investigated the effects of proton adsorption of the CCM segments under endosomal acidic pH on the size and morphology of CCM nanoassemblies by DLS and TEM analyses. Figure 3c shows the impacts of pH change on the size of CCM nanoassemblies based on DLS analyses. The size of CPC and PC_4_ nanoassemblies obviously increased with the decrease in pH from 7.4 to 5.5, while no changes were observed for PC and PCP nanoassemblies. Figure 3d shows TEM images of CPC and PC_4_ nanoassemblies incubated under pH 7.4 and pH 5.5, respectively. CPC and PC_4_ nanoassemblies at pH 7.4 had spherical and square-shaped morphology with average diameter of about 205 nm (CPC) and 79 nm (PC_4_), respectively. However, after incubation under pH 5.5, the diameter significantly increased up to 310 nm (CPC) and 450 nm (PC_4_), respectively. Moreover, the remarkable morphology change (swelling) of both CPC and PC_4_ nanoassemblies were observed, and uniformity in the size and the morphology was disordered. As mentioned above, CPC and PC_4_ nanoassemblies adsorb H^+^ ions to the CCM domains located inside of nanoassemblies, while PC and PCP nanoassemblies adsorb H^+^ ions at the near surface. Under the physiological environment (pH 7.4), most diketone groups of CCM segments in all types of nanoassemblies were deprotonated, and the hydrophobic interaction, π-π stacking, and hydrogen bonding between the CCM segments were the dominant forces that form condensed CCM domains to maintain nanoassemblies stable. When incubated at weak acidic pH, the hydrophobicity of CCM segments can be reduced due to the adsorption of H^+^ ions, leading to destabilization of the CCM domains. Furthermore, the cationic six-membered ring on protonated diketone groups in the CCM domains caused sensitive electrostatic repulsive force. These pH-responsive synergistic phenomena probably drove the swelling of nanoassemblies. It is reasonably understood that the destabilization of protonated CCM domains located inside of nanoassemblies (CPC and PC_4_) have great impact on the pH-responsive size and morphology change than that of CCM domains located at the near surface of nanoassemblies (PC and PCP). Interestingly, average size of PC_4_ nanoassembly gradually decreased when pH was reversely increased from 5.5 to 7.4, and the resultant nanostructures exhibited inherent square-shaped morphology with average diameter of about 115 nm and a narrow size distribution at pH 7.4 (Supplementary Fig. 10), indicating the reversible pH-responsiveness of PC_4_ nanoassemblies. It is well known that early endosome and matured endosomes (late endosomes and lysosomes) typically displayed diameters ranging from 100 to 200 nm and from 200 to 400 nm, respectively^25^. Thus, the size of CPC and PC_4_ nanoassemblies swelled at pH 5.5–6.5, corresponding to endosomal pH under the maturation process, was similar or larger than the size of endosomes, implying that the expansion force generated by the swelling of CPC and PC_4_ nanoassemblies can enhance their endosomal escaping activity.

### Endosomal escaping activity of CCM nanoassemblies

To assess the endosomal escaping activity of CCM nanoassemblies arising from the pH-responsive properties, damage of endosomal membrane was examined using acridine orange relocation assay^32^. Acridine orange is a pH-sensitive dye which diffuses into cells and accumulates in the acidic endosomes/lysosomes by proton trapping. The accumulation of acridine orange in acidic endosomes/lysosomes gives red-orange fluorescence, while acridine orange shows yellow-green fluorescence in neutral pH environment. Therefore, the rupture of endosomal/lysosomal membrane can be indicated by a shift of red-orange fluorescence to a yellow-green fluorescence in the presence of acridine orange. Figure 4a shows CLSM images of PC-3 cells treated with CCM nanoassemblies and free CCM in the presence of acridine orange, and Fig. 4b shows the time course of the average numbers of endosomes/lysosomes observed in a PC-3 cell based on the CLSM images. PC-3 cells treated with free CCM showed red-orange granules in the endosomes/lysosomes and a diffuse weak green fluorescence in the cytoplasm after 0.5 h. The numbers of endosomes/lysosomes slightly increased over time in the presence of free CCM, meaning that free CCM does not have endosome disrupting activity. PC-3 cells treated with CCM nanoassemblies revealed similar red-orange granules after 0.5 h, however the red-orange granules gradually reduced over time, indicating the expansion of endosomal/lysosomal membrane damage in the presence of CCM nanoassemblies. Thus, it was found that all types of CCM nanoassemblies have obvious endosomal/lysosomal membrane disrupting activity. The noticeable decrease in the red-orange granules is attributed to the burst release of acridine orange from endosomes/lysosomes to cytoplasm. Moreover, the following obvious increase in cytoplasmic green fluorescence intensity can be attributed to the accumulation of acridine orange as well as CCM nanoassemblies in the cytoplasm. Thus, it was clearly demonstrated that CCM nanoassemblies have effective endosomal escaping activity and sequential cytoplasmic self-delivering ability. Among them, CPC and PC_4_ nanoassemblies showed continuous decrease in the numbers of endosomes/lysosomes within 5 h, while increase of the numbers was started after 3 h for PC and PCP nanoassemblies. As mentioned above, only CPC and PC_4_ nanoassemblies exhibited remarkable pH-responsive size increase up to 350–450 nm which is similar or larger than the diameter of endosomes/lysosomes. Considering this, the pH-responsive size increase of CPC and PC_4_ nanoassemblies occurred within endosomes/lysosomes probably contribute to the endosomal escaping activity.

**Figure 5:**
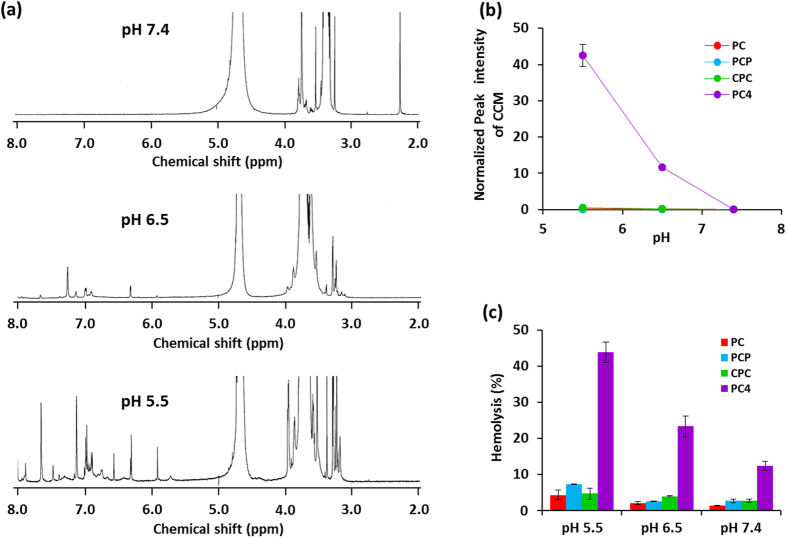
(**a**) ^1^H-NMR spectra of PC_4_ nanoassembly measured in D_2_O at pH 7.4, pH 6.5, and pH 5.5, respectively. (**b**) Relative peak intensity of curcumin in the nanoassembly measured in D_2_O at pH 5.5, pH 6.5, and pH 7.4, respectively. (**c**) pH-sensitive membrane-lytic activity of CCM nanoassemblies after 2 h of incubation with seep erythrocyte at pH 5.5, pH 6.5, and pH 7.4, respectively. Values are average of three separate experiments in triplicate and are expressed as mean ± SD.

In particular, only PC_4_ nanoassembly showed great decrease in the numbers of endosomes/lysosomes within 1.5 h, implying that PC_4_ nanoassembly have further additional mechanism for enhancement of the endosomal escaping activity. To obtain further information about pH-responsive properties of PC_4_ nanoassembly at molecular level, ^1^H-NMR spectra of PC_4_ nanoassembly were measured in D_2_O at different pH. As shown in Fig. 5a, the proton signal of PEG segment (3.4–3.8 ppm) was detected as a sharp peak, while the characteristic peaks of CCM segments (5.8–7.6 ppm) were completely disappeared at pH 7.4 because CCM segments form hydrophobic domains inside of the nanoassemblies and the major part of PEG segments extend out into aqueous environment at the surface of nanoassemblies. The peak intensity of CCM segments obviously increased with the decrease in pH from 7.4 to 5.5, indicating that the CCM domains were partially disrupted and the some parts of protonated CCM segments released from their domains were displayed at near the surface. This result is well consistent with the disordered CCM domains and swelled morphology of PC_4_ nanoassemblies at pH 5.5 detected by TEM observation. Importantly, such noticeable increase in the peak intensity of CCM segments was observed for only PC_4_ nanoassembly, as shown in Fig. 5b. Thus, it is plausible that the surface display of protonated CCM segments in response to weak acidic pH can be an additional source to enhance the endosomal escaping activity of PC_4_ nanoassembly. Barry and co-workers reported that CCM molecules anchored in cell membrane with high density caused disruption of the membrane structure^10^. Inspired by this report, pH-responsive membrane-lytic property of CCM-based nanoassemblies was further studied with erythrocytes as a model for the endosomal membrane^33^. As shown in Fig. 5c, membrane-lysis was not induced by treatments of PC, PCP, and CPC nanoassemblies under any pH conditions. By contrast, PC_4_ nanoassembly clearly showed membrane-lytic activity and the activity was enhanced at weak acidic pH condition (pH 6.5 and 5.5). Taken together, it was found that the membrane-lytic activity of PC_4_ nanoassembly is caused by the protonated CCM segments, displayed at the surface, which are capable of biding to the erythrocyte membrane through electrostatic interaction and subsequent insertion into the membrane, resulting in the disruption of cell membrane.

### *In vitro* cytotoxicity of CCM nanoassemblies

**Figure 6:**
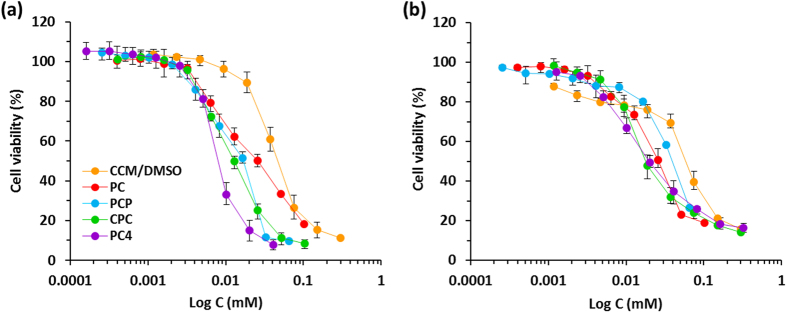
(**a**) Cell viability of (**a**) PC-3 cells and (**b**) HepG2 cells treated with CCM nanoassemblies for 24 h. Values are average of three separate experiments in triplicate and are expressed as mean ± SD.

To evaluate the potential of CCM nanoassemblies as anticancer nanodrugs, *in vitro* cytotoxicity was evaluated using cancer cell lines (PC-3 and HepG2 cells). As shown in Fig. 6, cancer cells treated with all types of CCM nanoassemblies (with the same concentration at CCM level) showed a typical dose-dependence sigmoidal curve. This result indicates that the cytotoxicity is derived from the CCM nanoassemblies, thus CCM nanoassemblies can act as anticancer nanodrugs. The half maximum inhibitory concentration (IC_50_) after 24 h were calculated from the obtained sigmoidal curves and the values were summarized in Supplementary Table 2. All types of CCM nanoassemblies showed lower IC_50_ values than free CCM for both PC-3 and HepG2 cells. Importantly, PC_4_ nanoassemblies showed the lowest IC_50_ value for PC-3 cell, and CPC and PC_4_ nanoassemblies showed the lowest IC_50_ values for HepG2 cell, indicating that the *in vitro* cytotoxicity depends on the endosomal escaping activity to deliver themselves into cytoplasm as the site of action of CCM for cytotoxicity.

### *In vivo* studies of anticancer CCM nanodrugs

**Figure 7:**
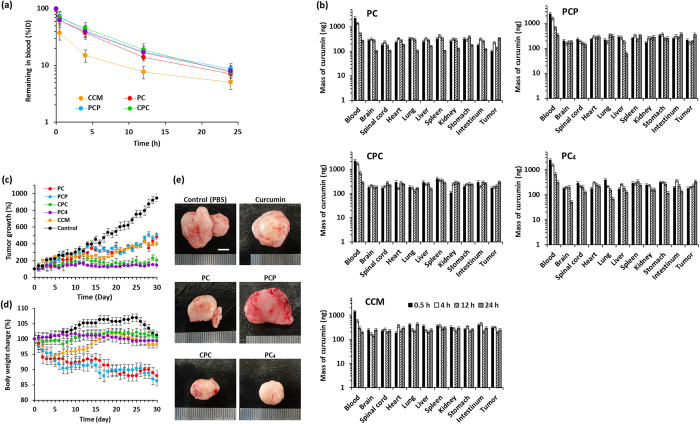
Biodistribution and *in vivo* antitumor efficacy of CCM nanoassemblies in tumor-bearing nude mice. (**a**) Blood concentration vs. time profiles of CCM nanoassemblies and free CCM after i.v. injection into tumor-bearing mice. The data were expressed as the percentage of CCM in blood against injected dose (%ID). (**b**) Tissue distribution of CCM nanoassemblies and free CCM after i.v. injection into tumor-bearing mice. Values are average of three separate experiments in triplicate and are expressed as mean ± SD. (**c**) Tumor volume change after the treatments. (**d**) The size of *ex vivo* tumor after 30 days treatments. (**e**) Mice bogy weight change during the treatment. Scale bars indicate 5 mm. Values are average of three separate experiments in triplicate and are expressed as mean ± SD.

Generally, nanoparticles with a suitable size (<250 nm) show a longer blood retention time as compared to free small-molecule drugs^34^. To evaluate the influences of supramolecular nanoassembly of CCM on the blood circulation profiles, tumor-bearing mice were treated with single intravenous injection of CCM nanodrugs or free CCM, collected plasma at different time intervals, and then estimated the plasma concentration at CCM level by UV-Vis measurements. As shown in Fig. 7a, the plasma concentration of free CCM sharply decreased to approximately 35% of the initial maximum dose within 0.5 h, indicating rapid clearance of free CCM from the circulation system. By contrast, all types of CCM nanodrugs showed much prolonged blood circulation time with significantly higher CCM concentration over the free CCM. To evaluate the biodistribution profiles, tumor-bearing mice treated with single intravenous injection of CCM nanodrugs or free CCM were sacrificed, and the amounts of CCM accumulated in major organs were estimated by UV-Vis measurements at 0.5 h, 4 h, 12 h, and 24 h post-injection. As shown in Fig. 7b, biased accumulation in specific organs was not observed for all types of nanodrugs. The amounts of CCM nanodrugs accumulated in reticuloendothelial systems, such as liver and spleen, and kidney which are responsible for active clearance of CCM from circulation, was almost similar with that of free CCM. This result indicates that the prolonged blood circulation time of CCM nanodrugs over free CCM would be mainly due to the improved hydrolysis resistance, as described in our previous report^23^. The amount of CCM nanodrugs accumulated in tumor tissues increased over time at least within 24 h. After 24 h, the amounts accumulated in tumor were reached to 316 ng (PC), 345 ng (PCP), 322 ng (CPC), and 334 ng (PC_4_), while that of free CCM was 238 ng, indicating higher tumor accumulation of CCM nanodrugs than free CCM owing to possible passive targeting, EPR effect.

To elucidate whether pH-responsive endosomal escaping activity of anticancer CCM nanodrugs results in the enhancement of anticancer therapeutic potency, PC-3 tumor-bearing mice were intravenously injected with CCM nanodrugs dispersed in PBS, free CCM dissolved in glycerol formal, or PBS only as negative control via the tail vein with volume at 2 mL/kg and an equivalent dose of CCM in nanodrugs at 10 mg/kg, respectively. Tumor volume and body weight of tumor-bearing mice were monitored every day for 30 days. Tumor growth was clearly inhibited after the treatment with free CCM and all types of CCM nanodrugs as compared with PBS treatment (Fig. 7c). The tumor inhibitory rate was calculated from tumor volume. Compared with tumor inhibitory rate of PBS group (100%) after 30 days, the inhibitory rate was 42.5% (free CCM), 51.1% (PC), 54.3% (PCP), 22.3% (CPC), and 12.8% (PC_4_), meaning that CPC and PC_4_ nanodrugs showed significantly better *in vivo* anticancer efficacy than free CCM and even PC and PCP nanodrugs. It is noteworthy that PC_4_ nanodrugs completely inhibited the tumor growth (the tumor volume was not changed during the treatment).

The *in vivo* undesired toxicity arising from nano-sized materials which can cause harmful side effects has usually been one of major concerns in the development of nanomedicines^35^. Therefore, the body weight change of tumor-bearing mice treated with CCM nanodrugs or free CCM with the same dose at CCM level was examined. As shown in Fig. 7d, there is about 7% loss of body weight for mice treated with PC and PCP nanodrugs and free CCM after 5 days. After that, the weight loss was stopped and gradual increase was observed for free CCM-treated mice, while the weight loss was continuously caused and reached to about 15% after 30 days for PC and PCP nanodrug treatments, indicating side effects of PC and PCP nanodrugs for tumor therapy. By contrast, no weight loss was observed for mice treatment with CPC and PC_4_ nanodrugs.

Formation of tumor-associated neovascular networks has usually been one of major concerns in tumor metastasis. Indeed, it has been reported that CCM inhibits angiogenesis *in vitro* through down-regulation of the expression of proangiogenic genes, such as VEGF, angiopoietin 1 and 2^36^. To elucidate the inhibition of tumor-associated angiogenesis by CCM nanodrug treatments, mice were sacrificed and tumors were separated at the end of experiments. Although obvious neovascular networks were generated at the tumor surface for PC, PCP, and CPC nanodrugs and free CCM treatments, PC_4_ nanodrugs completely inhibited tumor-associated neovascularization (Fig. 7e), suggesting a potential inhibitory effect on tumor metastasis of PC_4_ nanodrug. All these results from *in vivo* experiments demonstrated that CPC and PC_4_ nanodrugs have obvious anticancer potency without harmful side effects available for anticancer therapy.

### Hybrid anticancer nanodrugs fabrication

Combination therapy which uses two or more kinds of drugs with different mechanism of action has recently gained much attention as new trend in the field of nanomedicines. Therefore, we evaluated the potential utility of CPC and PC_4_ nanodrugs as delivery carriers for combination therapy. As a combination drug with CCM, we selected doxorubicin (DOX), a common anthracycline antibiotic. DOX interacts with DNA in cell nucleus through the intercalation into DNA double helix, leading to inhibition of the DNA synthesis or poisoning of topoisomerase II^37^. DOX is used for treatment of various cancers, especially breast, ovarian, prostate, brain, lung, and leukemia^38^,^39^. However, the anticancer efficacy of DOX is often compromised by multidrug resistance mechanisms involving P-gp proteins. DOX is a substrate for drug transporters such as of Multidrug Resistance 1 (MDR1) and Multidrug Resistance-Related Protein 1 (MRP1)^38^. Furthermore, DOX is a weak base with a p*K*_a_ near neutrality; it is prone to accumulating in acidic cytoplasmic vesicles of cancer cells by active transport mechanism, thereby forming sink conditions in the cytosol of cancer cells^39^. These drawbacks lead to decreased sensitivity of DOX to cancer cells^40^. To overcome the drawbacks of DOX, high cellular uptake and subsequent cytoplasmic delivery via pH-responsive endosomal escaping of CPC and PC_4_ nanodrugs can be helpful. Moreover, synergistic anticancer effects can be expected because CCM and DOX have different anticancer mechanism. Therefore, we evaluated a potential utility of CPC and PC_4_ nanodrugs as DOX delivery carriers. From the viewpoint of molecular structure, DOX has π-conjugated framework and multiple hydroxyl groups (Supplementary Fig. 11c) capable of generating π-π stacking interaction and hydrogen bonding with CCM molecule. Considering this, we attempted to fabricate supramolecular co-assembled hybrid nanodrugs consisting of CCM and DOX. In this study, molar ratio of CCM amphiphile (100 μM at CCM level) and DOX (50 μM) was fixed at 2:1. CPC or PC_4_ molecules were dissolved in aqueous DOX solution and then sonicated to give co-assembled hybrid nanodrugs. Supplementary Fig. 12 shows DLS data of CPC/DOX and PC_4_/DOX hybrid nanodrugs, and free DOX. Both CPC/DOX and PC_4_/DOX hybrid nanodrugs showed a unimodal peak with narrow size distribution, and free DOX peak was not detected in the presence of CPC and PC_4_, revealing the formation of co-assembled hybrid nanodrugs with well-defined size. The average diameter was 182 nm (CPC/DOX hybrid) and 73 nm (PC_4_/DOX hybrid), respectively. The architecture of CCM, DOX, and PEG in the hybrid nanodrugs at molecular level was analyzed by ^1^H-NMR measurement in D_2_O. Supplementary Fig. 11a,b shows ^1^H-NMR spectra of CPC/DOX and PC_4_/DOX hybrid nanodrugs. The proton signals of CCM segments and DOX were not detected but the proton signal of PEG segment was detected as a sharp peak. This result indicates that hybrid domains consisting of CCM segments and DOX were formed inside of hybrid nanodrugs and the major part of PEG segments were extended out into aqueous environment at the surface of hybrid nanodrugs.

**Figure 8:**
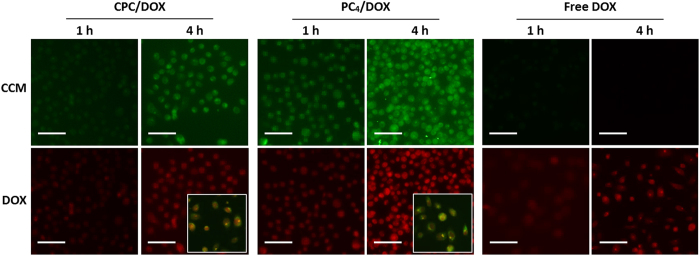
Fluorescence microscope images of PC-3 cells treated with CCM/DOX hybrid nanoassemblies after 1 h and 4 h of treatment. Green: CCM nanoassemblies, red: doxorubicin. Scale bars indicate 100 μm. The insets represent the merged images of PC-3 cells treated with CCM/DOX hybrid nanoassemblies for 4 h. The yellow signal indicates CCM/DOX hybrid nanoassemblies.

Cellular uptakes and intracellular trafficking of the CCM/DOX hybrid nanodrugs were investigated using PC-3 cancer cells. As shown in Fig. 8, both green (CCM-based nanodrugs) and red (DOX) fluorescence intensity in cytoplasm of PC-3 cells treated with CPC/DOX and PC_4_/DOX hybrid nanodrugs significantly increased over time, and the red fluorescence intensity in cytoplasm was much higher than that with free DOX treatment, revealing that CPC and PC_4_ nanodrugs have a potential as cytoplasmic delivery carriers for DOX. Importantly, the red fluorescence intensity in cells treated with PC_4_/DOX hybrid nanodrugs was much higher than CPC/DOX hybrid nanodrugs, indicating that the superior endosomal escaping activity of PC_4_ nanodrugs was still functioning in PC_4_/DOX hybrid nanodrugs. Note that, yellow fluorescence and green fluorescence signals were detected in cytoplasm and red fluorescence signal was detected in nucleus according to the merged images, indicating that the hybrid nanodrugs were effectively delivered to cytoplasm, and then DOX was released into cytoplasm. Most importantly, CPC/PC_4_ nanodrugs stayed in the cytoplasm as the site of action of CCM, while released DOX entered into the nucleus as their action site. Thus, the CCM/DOX hybrid systems can overcome the drawbacks of DOX, and thus both CCM and DOX could exhibit inherent anticancer activity.

## Discussion

**Figure 9:**
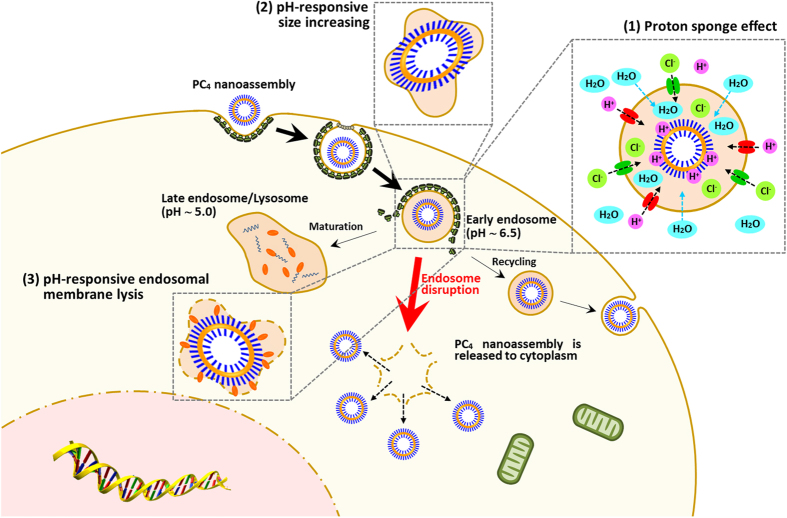
Schematic illustration of endosomal escape of PC_4_ nanoassembly facilitated by curcumin segments-based (1) proton sponge effect, (2) pH-responsive size increasing effect, and (3) pH-responsive endosomal membrane-lytic activity after cellular internalization.

Based on the experimental results described above and the facts reported previously, mechanism of pH-responsive endosomal escape of CCM nanoassemblies was proposed. It is well known that polymer-based carrier nanomaterials with high pH buffering capacity, such as polyethylenimine, polyhistidine, and polyamidoamine dendrimer, perform endosomal escaping activity via proton sponge effect^41^,^42^,^43^,^44^,^45^. Since CCM nanoassemblies also have effective pH buffering capacity, proton sponge effect can be one of potential mechanisms for the endosomal escaping activity. CCM nanoassemblies are entered into cancer cell through clathrin-mediated endocytosis and trapped in acidic endosomes. The CCM segments in nanoassemblies become protonated by H^+^ ion adsorption under endosomal acidic pH condition. H^+^ ions are further supplied by the V-ATPase (proton pump) during the endosomes maturation. This process keeps the pump functioning and leads to the influx of one Cl^−^ ion and one water molecule per proton^46^. The influx of Cl^−^ ions and water molecules into the endosomes/lysosomes caused an increase in osmotic pressure and tension on the endosomal/lysosomal membrane, which subsequently induces the endosomes/lysosomes swelling and possible disruption of the membrane, resulting in the release of CCM nanoassemblies to cytoplasm. CPC and PC_4_ nanoassemblies with higher buffering capacity induce more influx of Cl^−^ ions and water molecules into the endosomes/lysosomes, generating higher osmotic pressure which facilitates the endosomal escape. For PC and PCP nanoassemblies, the increased osmotic pressure and the related tension arising from proton sponge effect within endosomes/lysosomes is possible mechanism for their endosomal escaping activity (Supplementary Fig. 13). In addition to the osmotic pressure and internal tension, expansion force arising from the pH-responsive size increase (swelling) generated within endosomes/lysosomes are possible endosomal escaping mechanism for CPC nanoassemblies (Supplementary Fig. 14). Thus, the synergistic mechanism facilitates the endosomal escaping activity of CPC nanoassemblies. In case of PC_4_ nanoassemblies, the increased osmotic pressure and internal tension, expansion force arising from the pH-responsive size increase, as well as pH-responsive membrane-lytic activity are possible endosomal escaping mechanism (Fig. 9). Thus, the synergistic and sequential multiple mechanism significantly enhances the endosomal escaping activity of PC_4_ nanoassemblies. Impotrantly, the *in vivo* anticancer activity of CCM nanodrugs is in accordance with the results of *in vitro* cytotoxicity and endosomal escaping activity. As mentioned above, there was no significant difference in the tumor accumulation between four types of CCM nanodrugs. Considering the results, the superior *in vivo* anticancer activity of PC_4_ nanodrugs can be attributed to the highly effective pH-responsive endosomal escaping activity and subsequent cytoplasmic self-delivering ability. Thus, it is demonstrated that the pH-responsive endosomal escaping activity of CCM, newly discovered in this study, directly results in enhancement of their anticancer potency.

In summary, we have developed CCM nanoassemblies by adopting supramolecular self-assembly approach. Due to the pH-responsive endosomal disrupting activity of CCM nanoassemblies newly discovered in this study, the CPC and PC_4_ nanoassemblies performed endosomal escape via synergistic and sequential mechanisms. Importantly, systemic administration of CCM nanoassemblies with high endosomal escaping activity showed superior *in vivo* anticancer efficacy than nanoassemblies with low endosomal escaping activity and CCM alone. Thus, we demonstrated an important contribution of the pH-responsive endosome disrupting activity of CCM to enhance its anticancer activity. Furthermore, we demonstrated an additional usable ability of CCM nanodrugs as cytoplasmic delivery carriers for DOX toward combination cancer nanomedicine. Overall, this study can powerfully move forward with a clinical application of CCM and open new opportunity of CCM as biomaterials in nanomedicines.

## Methods

### Materials

Curcumin was purchased from Tokyo Chemical Industry (Tokyo, Japan). Poly(ethylene glycol) methyl ether (MePEG750, *M*_w_: 750 Da), poly(ethylene glycol)-diol (PEG1500, *M*_w_: 1500 Da) and 4-arm branched poly(ethylene glycol) (4-arm PEG, *M*_w_: 10,000 Da) were purchased from Sigma-Aldrich Japan (Tokyo, Japan). 4-Dimethylaminopyridine (DMAP) and *N*,*N′*-carbonyldiimidazole (CDI), dehydrated tetrahydrofuran (THF), dehydrated dichloromethane, diethyl ether, dimethyl sulfoxide (DMSO), methanol, deuterated chloroform (containing TMS), and deuterated water were purchased from Wako Pure Chemical Industries (Osaka, Japan). Dialysis membrane Spectra/Por 7 (MWCO: 3500) was purchased from Spectrum Laboratories. HepG2 human hepatocellular liver carcinoma cell and PC-3 human prostate cancer cell were purchased from the American Type Culture Collection (ATCC). 3-(4,5-dimethylthial-2-yl)-2,5-diphenyltetrazalium bromide (MTT) and Hoechst 33342 were purchased from Dojindo (Kumamoto, Japan).

### Synthesis of CCM amphiphiles

MePEG750 (PC: 308 mg, 0.41 mmol, PCP: 615 mg, 0.82 mmol), PEG1500 (CPC: 308 mg, 0.21 mmol), or (4-arm PEG: 200 mg, 0.020 mmol) were dissolved in 3 mL of THF (for PC, PCP, and CPC) or dichloromethane (for PC_4_) at room temperature. The obtained PEG solution was added into 4 mL of THF solution containing CDI (PC: 100 mg, 0.62 mmol; PCP and CPC: 200 mg, 1.23 mol; and PC_4_: 20 mg, 0.12 mmol) and DMAP (PC: 50 mg, 0.41 mmol; PCP: 100 mg, 0.82 mmol; CPC: 50 mg, 0.42 mmol; and PC_4_: 10 mg, 0.080 mmol), and then stirred at room temperature for 6 h. After that, the resultant solution was added dropwise into 5 mL of THF solution containing CCM (PC, PCP, and CPC: 150 mg, 0.41 mmol; and PC_4_: 30 mg, 0.08 mmol) and DMAP (PC and CPC: 100 mg, 0.82 mmol; PCP: 150 mg, 1.23 mmol, and PC_4_: 80 mg, 0.64 mmol), and stirred at room temperature for 40 h. The reaction solution was added dropwise into 250 mL of cold diethyl ether and stirred for 120 min to give precipitate of CCM-PEG conjugates. Diethyl ether dissolving non-reacted CCM and reaction byproducts was removed by centrifugation (3000 rpm for 3 min) and the precipitate was washed by cold diethyl ether twice, and then the precipitate was dried under vacuum for 48 h to give yellow flake of CCM-PEG conjugates. Characterization of the obtained CCM amphiphiles were carried out by ^1^H-NMR measurement (JEOL, ECA-500, solvent: CDCl_3_) and gel permeation chromatography (GPC) (JASCO, HPLC LC-2000Plus, column: TSKgel^®^, detector: RI, standard: PEG, eluent: DMSO dissolving 10 mM LiBr).

### Preparation and characterization of CCM nanoassemblies

The CCM amphiphiles were directly dissolved in pure water at room temperature and the solution was sonicated for 10 min to give their self-assembled nanostructures. The average diameters of nanoassemblies in pure water were measured by dynamic light scattering (DLS, ZETASIZER NanoSeries ZEN-3600, Malvern). The critical assembly concentration (CAC) of CCM-PEG conjugates in pure water was analyzed by UV-Vis spectroscopy using eosin Y as probe molecule at 20 °C. Eosin Y solution (final concentration: 20 μM) was added to CCM-PEG conjugate solution and incubated for 2 h, and then the absorbance of eosin Y at 518 nm was measured by UV-Vis spectrophotometer (JASCO, V-630). The shape of nanoassemblies was visualized by transmission electron microscope (TEM) observation (JEOL, JEM-1400). One drop of aqueous nanoassembly solution (concentration: 100 μM) was carefully placed on a copper grid, air-dried under vacuum prior to TEM observation. The morphology of CCM nanoassemblies was analyzed by ^1^H-NMR measurement in D_2_O.

### Cellular uptake

PC-3 cell were cultured in DMEM supplemented with 10% heat-inactivated FBS, 0.15% NaHCO_3_, 2 mM L-glutamine, 100 U/mL penicillin, 100 μg/mL streptomycin and the culture was maintained in a humidified incubator at 37 °C with 5% CO_2_. PC-3 cells (5.0 × 10^4^ cells) were seeded on 24-well cell culture plate and incubated for 24 h. Cellular transport inhibitors (chlorpromazine hydrochloride: 40 μM, genistein: 40 μM, cytochalasin D: 4 μM) were added to medium and incubated for 2 h, and medium was freshly changed prior to addition of CCM nanoassemblies (200 μM). After 1, 2, 4, and 6 h incubation with the nanoassemblies, 100 μL of medium containing nanoassemblies was taken and analyzed by UV-Vis spectroscopy at 430 nm, corresponding to λ_max_ of CCM, to estimate the amount of CCM nanoassemblies in medium. PC-3 cells treated with nanoassemblies for 6 h were observed by fluorescence microscopy measurements. The nuclei were stained with Hoechst 33342. PC-3 cells (2.5 × 10^5^ cells) were seeded on glass bottom cell culture dish (35 mm) and incubated for 24 h prior to addition of CCM nanoassemblies (100 μM). After 1, 4, 8, and 12 h incubation with the nanoassemblies, supernatant was removed and cells were washed gently with PBS twice, and then fresh medium was added to the dish. Intracellular distribution of CCM nanoassemblies was observed by fluorescence microscopy measurements. Late endosomes and lysosomes were stained with LysoTracker Red and the nuclei were stained with Hoechst 33342.

### Acid-base titration

The buffering capacity of CCM nanoassemblies was analyzed by an acid-base titration method. CCM nanoassemblies were directly prepared in 10 mM NaOH solution and the solution pH was adjusted to pH 9.0 by addition of 10 mM NaOH solution to obtain the nanoassembly solution of 200 μM in CCM level. Thus, the CCM concentration of PC, PCP, CPC, and PC_4_ nanoassembly solution was the same. Fifty μL of 2 mM HCl was added to the solution and the pH value was measured. This titration procedure was continued at pH 3.5. As a control, buffering capacity of CCM was also analyzed by the same method.

### pH-responsive size, morphology, and zeta potential changes of CCM nanoassemblies

CCM-PEG conjugates were directly prepared in pH-controlled water (pH 5.5, 6.0, 6.5, and 7.4) at 100 μM concentration, and pH was finally adjusted to 5.5, 6.0 6.5, or 7.4 by addition of 2 mM HCl or 10 mM NaOH solution. The size and zeta potential of the obtained CCM nanoassembly solutions were analyzed by DLS with capability to measure surface zeta potential. The morphology of nanoassemblies at each pH was analyzed by TEM observation. One drop of aqueous nanoassembly solution at various pH was carefully placed on a copper grid, air-dried under vacuum prior to TEM observation. The morphology of CCM nanoassemblies at molecular level in D_2_O (200 μM) at different pH (5.5, 6.5, and 7.4) was analyzed by ^1^H-NMR measurement.

### Endosomal escape of CCM nanoassemblies

Endosomal escaping activity of CCM nanoassemblies was assessed using acridine orange method. PC-3 cells (3.0 × 10^4^ cells) were seeded on glass bottom 8-well plate and incubated for 24 h prior to addition of CCM nanoassemblies (100 μM). After 0.5, 1.5, 3.0, and 5.0 h incubation with the nanoassemblies, acridine orange (3.0 μg/mL) solution in PBS was added to medium and incubated for 20 min. After that, medium was removed and cells were washed gently with PBS twice, followed by fluorescence microscope observation.

### pH-dependent hemolytic activity of CCM nanoassemblies

The pH-responsive membrane-lytic activity of CCM nanoassemblies was assessed using erythrocytes as a model of the endosomal membrane. EDTA-treated sheep whole blood (7 mL) was centrifuged for 5 min at 1600 rpm and the resultant pellet was washed several time with PBS (pH 7.4) until the supernatant was clear and colorless. Finally, 10 mL of erythrocyte suspension was prepared by PBS (pH 7.4). Erythrocyte suspension (180 μL) was seeded into a 96-well plate followed by centrifugation (1600 rpm, 5 min) to obtain pellets of erythrocyte in each well. The aqueous CCM nanoassembly solution (200 μM) at pH 5.5, 6.5, and 7.4 was added to each well, and the pellet was gently resuspended with the sample solutions. After 2 h incubation with the nanostructures at 37 °C, 96-well plate was centrifuged at 4000 rpm for 5 min. The supernatant (100 μL) was transferred to new 96-well plate and the absorbance at 540 nm was measured by microplate reader (Bio-Rad, iMark) to determine the released hemoglobin. Two controls were prepared by resuspending erythrocyte either in PBS alone with pH 5.5, 6.5, and 7.4 (negative control) or in pure water (positive control). The percentage of hemolysis for CCM nanoassemblies was estimated by comparing the absorbance of samples with that of positive control.

### *In vitro* cytotoxicity of CCM nanoassemblies

Cytotoxicity induced by CCM nanoassemblies was investigated by conventional MTT assay. 200 μL of cell suspension (PC-3 and HepG2) was seeded to 96-well plate (1.0 × 10^4^ cells) and incubated for 24 h in a humidified incubator at 37 °C with 5% CO_2_. PBS solutions of nanoassemblies with defined concentrations were added to each well and incubated for 24 h. Then, 10 μL of MTT solution was added to each well and incubated for 4 h. The supernatant was removed and MTT-formazan crystals formed in cells were dissolved by addition of 200 μL of isopropyl alcohol containing 0.04 M HCl and 10% Triton-X, and the absorbance at 570 nm was measured by microplate reader.

### *In vivo* distribution of CCM nanoassemblies

All animal experiments and all experimental protocols were approved by Konan University (protocol No.: K-13-06), and conformed to the *Guidelines for the Care and Use of Laboratory Animals* published by the National Institutes of Health.

Female nude mice (BALB/cSlc-nu/nu) at 6 weeks of age were purchased from Charles River. For surgery, the animals were anesthetized using isoflurane and the surgical area was cleaned. PC-3 cells (2.0 × 10^6^ cells) in 200 μL of Matrigel (BD Bioscience) were injected subcutaneously into the back bilaterally using a disposable syringe and a 26-gauge needle. When the tumor size reached 200 mm^3^, animals were randomly divided into 6 groups (PBS only, free CCM, PC, PCP, CPC, and PC_4_, n = 3). CCM nanoassemblies dispersed in PBS or free CCM dissolved in glycerol formal were injected to tumor-bearing mice intravenously through the tail vein with volume at 2 mL/kg and an equivalent dose of CCM in each nanoassembly at 10 mg/kg, respectively. Then, 0.5, 4, 12, and 24 h after administration, the mice was sacrificed and tumor tissue, blood, and main organs (brain, spinal cord, heart, lung, liver, spleen, kidney, stomach, and intestine) were excised carefully for quantitative analysis of the accumulated CCM. These tissues and organs were homogenized and 500 μL of mixed solution (DMSO/MeOH = 1/4, v/v) was added followed by centrifugation at 4000 rpm for 30 min. Then, the supernatant was measured by UV-Vis spectroscopy at 430 nm, corresponding to λ_max_ of CCM, to estimate the amount of CCM nanoassemblies in these tissues and organs.

### *In vivo* antitumor activity of CCM nanoassemblies

Female nude mice (BALB/cSlc-nu/nu) at 6 weeks of age were anesthetized using isoflurane and the surgical area was cleaned. PC-3 cells (2.0 × 10^6^ cells) in 200 μL of Matrigel (BD Bioscience) were injected subcutaneously into the back bilaterally using a disposable syringe and a 26-gauge needle. When the tumor size reached 200 mm^3^, animals were randomly divided into 6 groups (PBS only, free CCM, PC, PCP, CPC, and PC_4_, n = 3). CCM nanoassemblies dispersed in PBS or free CCM dissolved in glycerol formal were injected to tumor-bearing mice intravenously through the tail vein with volume at 2 mL/kg and an equivalent dose of CCM in each nanoassembly at 10 mg/kg, respectively. These samples were administrated thrice a week for 4 weeks. The tumor volume was measured everyday using caliper and calculated according to the formula: tumor volume = (shorter diameter)^2^ × (longer diameter)/2. Body weight of the mice was recorded every day. At the end of the study, tumor tissues were carefully excised and weighted.

### Co-assembly of CCM amphiphiles with DOX

CCM amphiphiles (CCM concentration: 100 μM) were dissolved in aqueous DOX solution (50 μM) at room temperature and the solution was sonicated for 10 min to give co-assembled hybrid nanostructures composed of CCM amphiphiles and doxorubicin. The average diameters of the hybrid nanoassemblies were measured by DLS. The morphology of the hybrid nanoassemblies was analyzed by ^1^H-NMR measurement in D_2_O.

### Cellular uptake of CCM/DOX hybrid nanoassemblies

PC-3 cells (3.0 × 10^4^ cells) were seeded on glass bottom 8-well plate and incubated for 24 h prior to addition of CCM/DOX hybrid nanoassemblies (100 μM/50 μM). After 1 h and 4 h incubation with the hybrid nanoassemblies, medium was removed and washed gently with PBS twice, and then fresh medium was added to each well. Free DOX (50 μM) was used as negative control sample. Cell uptake and intracellular distribution of hybrid nanoassemblies were observed by fluorescence microscopy measurements.

## Additional Information

**How to cite this article**: Nagahama, K. *et al*. Discovery of a new function of curcumin which enhances its anticancer therapeutic potency. *Sci. Rep.* **6**, 30962; doi: 10.1038/srep30962 (2016).

## Supplementary Material

Supplementary Information
