# Supplementary material for: Discovery of a new function of curcumin which enhances its anticancer therapeutic potency
Source: Sci Rep. 2016 Aug 1;6:30962. doi: 10.1038/srep30962 (PMC4967984; doi:10.1038/srep30962)
Supplement: Supplementary Information [file srep30962-s1.pdf]

Supplementary Information for

Discovery of a new function of curcumin which enhances its anticancer therapeutic potency

Koji Nagahama,* Tomoya Utsumi, Takayuki Kumano, Saeko Maekawa, Naho Oyama & Junji Kawakami

Content:

Figure 1. Synthesis of CCM amphiphiles.

Figure 2-5. ^{1}H-NMR spectra of CCM amphiphiles measured in $CDCl_3$ and the nanoassembly measured in D_2O.

Figure 6. DLS profiles and TEM images of CCM nanoassemblies.

Figure 7. Fluorescence microscope images of PC-3 cells treated with free CCM.

Figure 8. Keto-enol tautomeric forms of curcumin.

Figure 9. Acid-base titration profiles of CCM amphiphiles in water/MeOH (7/3, v/v).

Figure 10. DLS profiles and TEM images of PC_4 nanoassembly in water at different pH values.

Figure 11. ^{1}H-NMR spectra of CCM/DOX hybrid nanoassemblies measured in D_2O.

Figure 12. DLS data of doxorubicin, and CCM/DOX hybrid nanoassemblies.

Figure 13, 14. Schematic illustration of endosomal escape of CCM nanoassemblies.

Table 1. Characterization of CCM amphiphiles.

Table 2. IC_{50} of the CCM nanoassemblies and CCM against cancer cells.

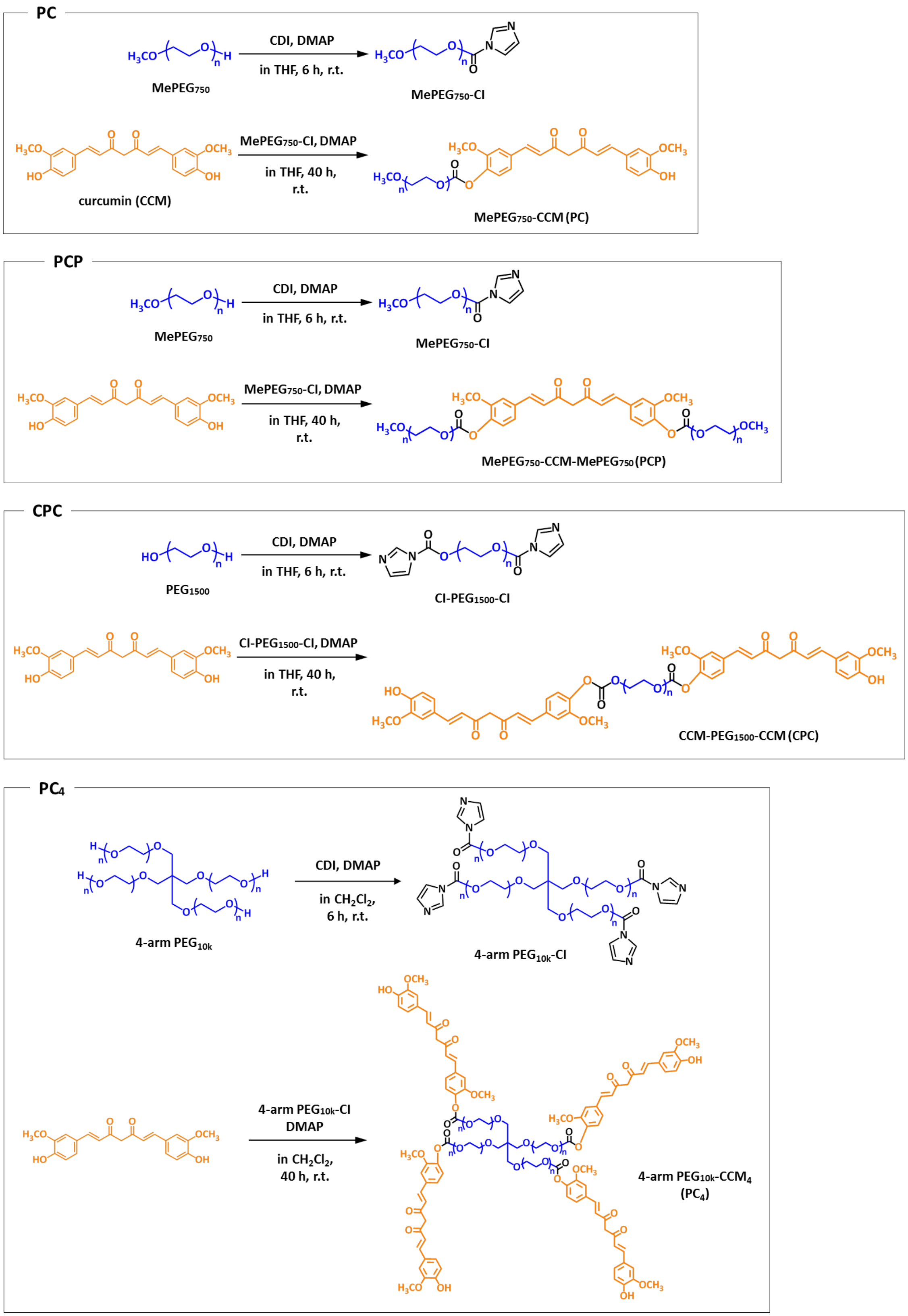

Figure 1. Synthesis of CCM amphiphiles synthesized in this study.

Table 1. Characterization of CCM amphiphiles

sample	structure	M_w, total[a)]	PD[b)]	PEG (%) substitution[c)]	CCM (%) content[d)]	CAC[e)] (M)
PC	PEG-CCM	1,140	1.17	99	32	1.14×10^{-6}
PCP	PEG-CCM-PEG	1,920	1.35	98	19	2.60×10^{-6}
CPC	CCM-PEG-CCM	2,290	1.23	98	32	4.80×10^{-7}
PC_4	4-arm PEG-CCM_4	11,580	1.31	97	13	3.54×10^{-6}

[a)]M_w of CCM amphiphiles were estimated from ^{1}H-NMR spectra measured in $CDCl_3$. [b)]Polydispersity of CCM amphiphiles were measured by GPC. [c)]PEG substitution of the conjugates were estimated from ^{1}H-NMR spectra measured in $CDCl_3$. [d)]Calculated by following equation: CCM content (%) = M_w of CCM segments/M_w of CCM amphiphile. [e)]Critical assembly concentration (CAC) of CCM amphiphiles in water.

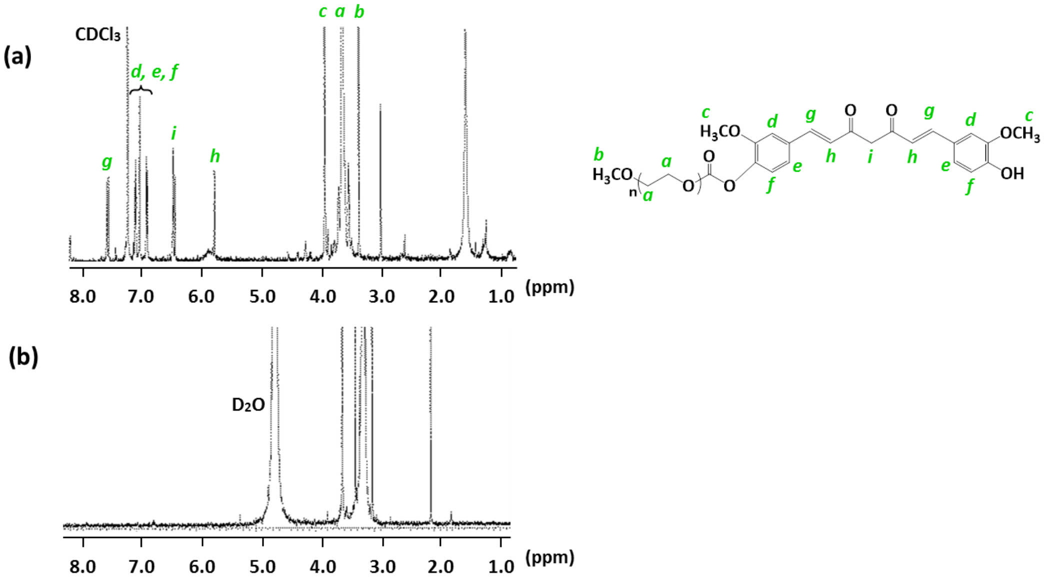

Figure 2. ^{1}H-NMR spectra of CCM amphiphile (PC) measured in $CDCl_3$ (a) and the nanoassembly measured in D_2O (b).

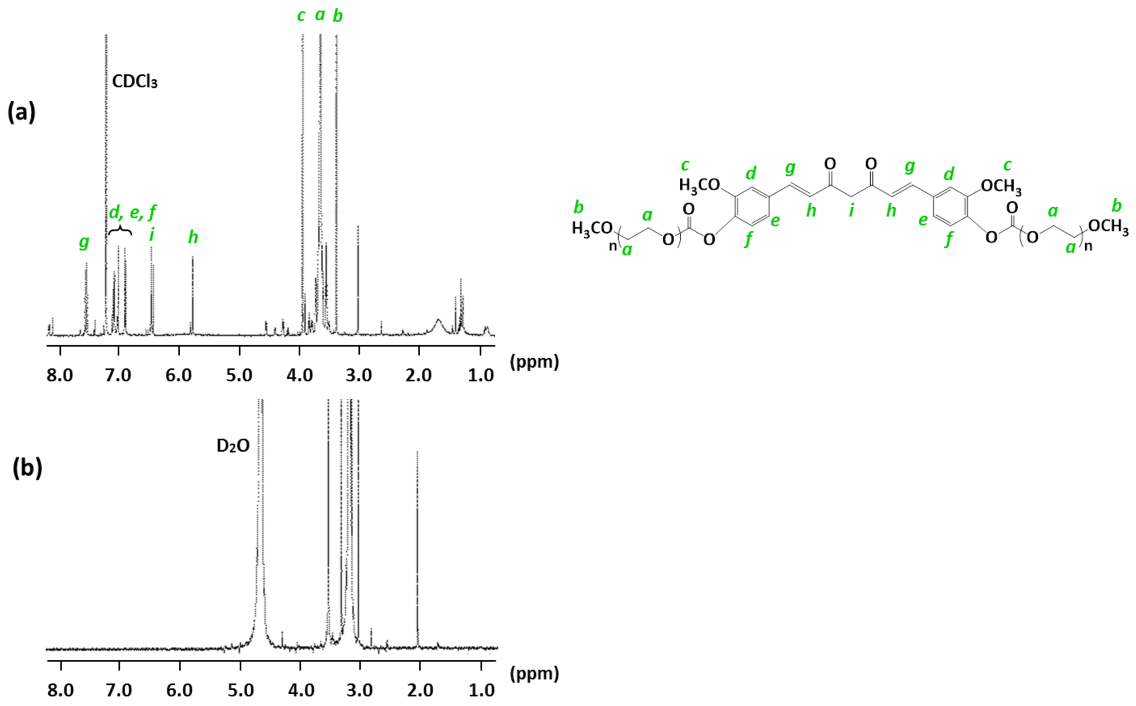

Figure 3. ^{1}H-NMR spectra of CCM amphiphile (PCP) measured in $CDCl_3$ (a) and the nanoassembly measured in D_2O (b).

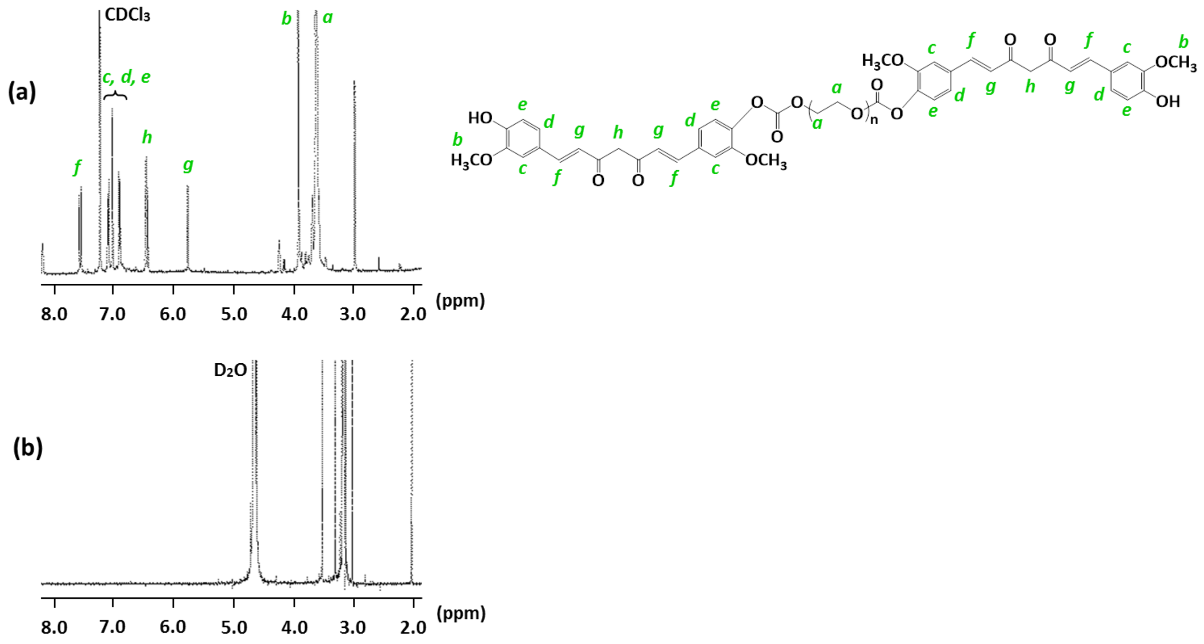

Figure 4. ^{1}H-NMR spectra of CCM amphiphile (CPC) measured in $CDCl_3$ (a) and the nanoassembly measured in D_2O (b).

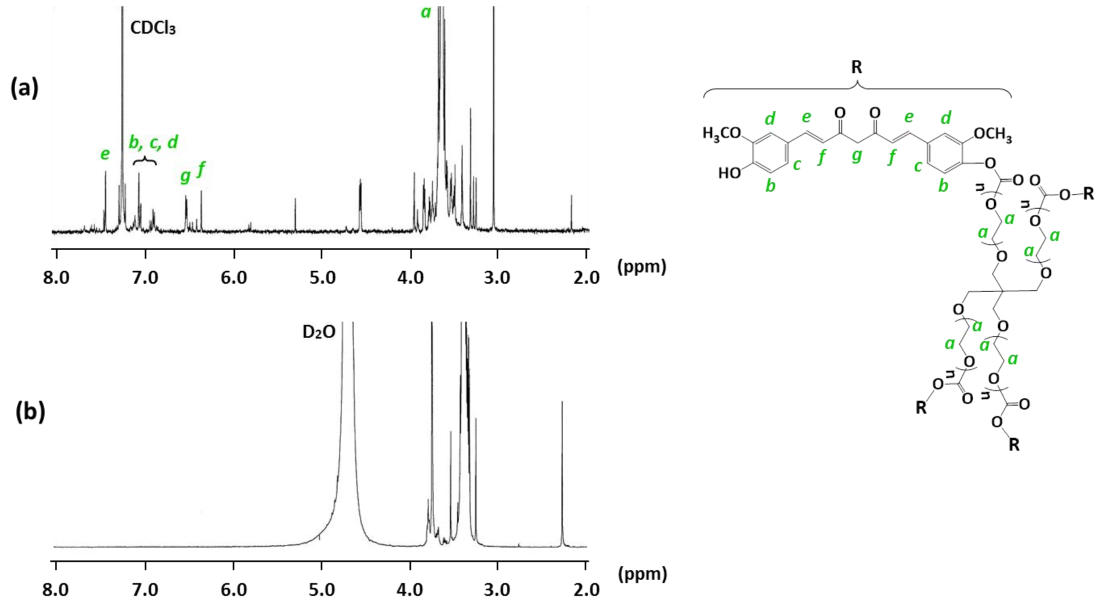

Figure 5. ^{1}H-NMR spectra of CCM amphiphile (PC_4) measured in $CDCl_3$ (a) and the nanoassembly measured in D_2O (b).

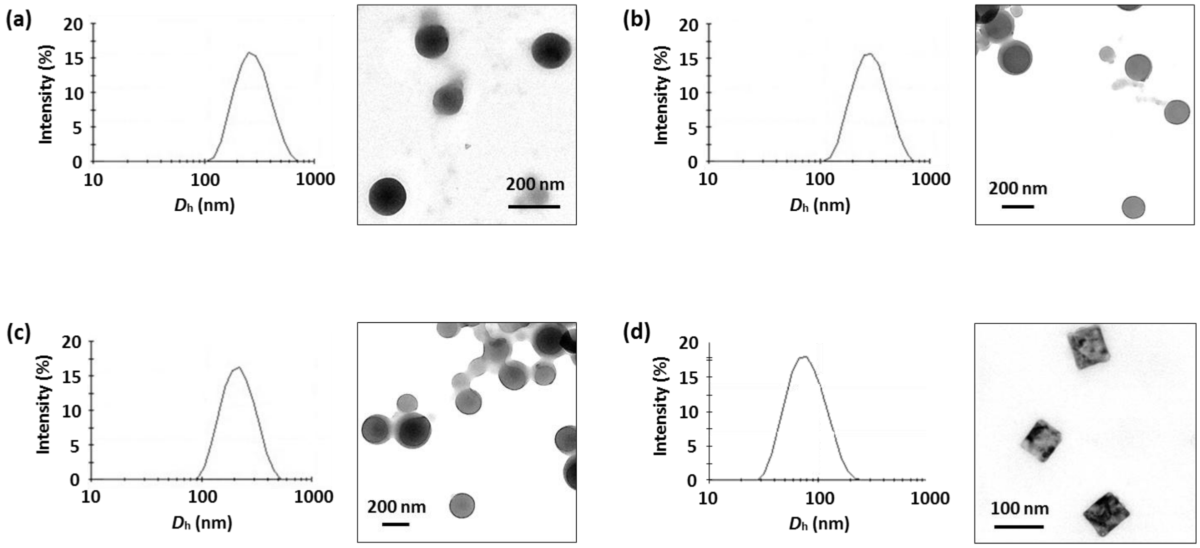

Figure 6. DLS profiles and TEM images of CCM nanoassemblies formed in water.

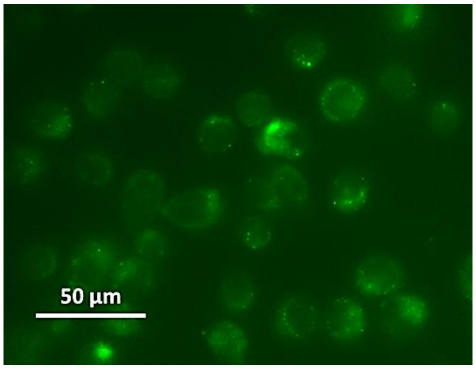

Figure 7. Fluorescence microscope images of PC-3 cells treated with free CCM.

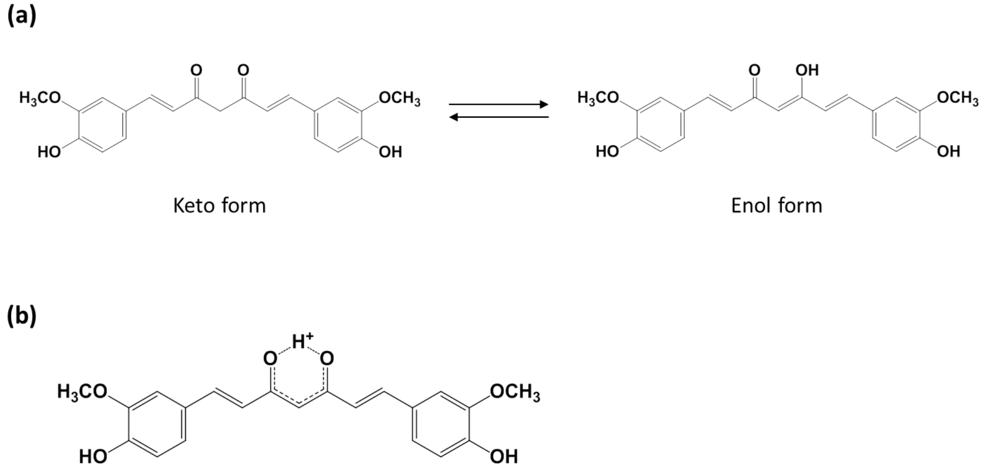

Figure 8. (a) Keto-enol tautomeric forms of curcumin. (b) Ketone form of protonated curcumin.

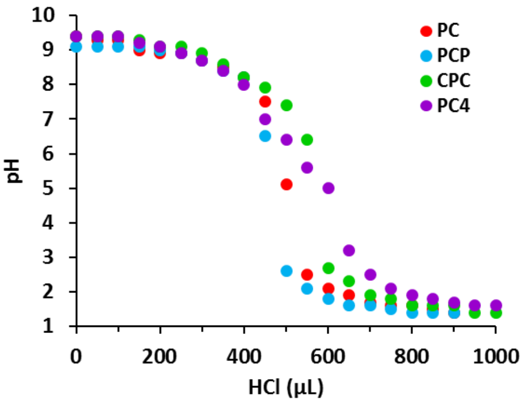

Figure 9. Acid-base titration profiles of CCM amphiphiles in water/MeOH (7/3, v/v) mixed solutions at 37 °C.

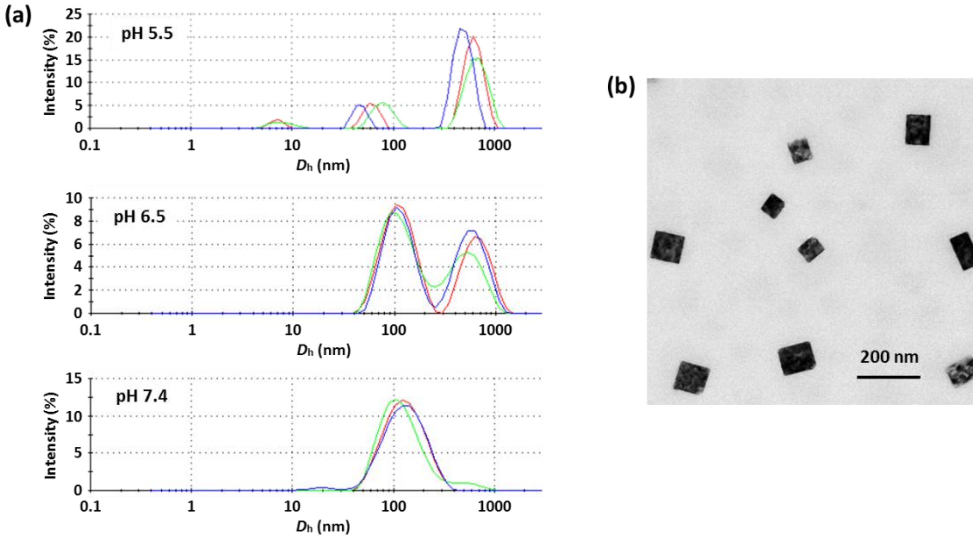

Figure 10. (a) DLS profiles of PC_4 nanoassembly in water at different pH values. pH was changed from 5.5 to 7.4 gradually by addition of aqueous NaOH solution. Each DLS data of three separate experiments are shown as different colored line. (b) TEM images of PC_4 nanoassembly incubated at pH 7.4 for 2 h.

Table 2. IC_{50} of the CCM-based nanoassemblies and CCM against cancer cells

sample	IC_{50} (M)[a)]	
	PC-3	HepG2
PC	2.8×10^{-5}	2.7×10^{-5}
PCP	1.8×10^{-5}	4.1×10^{-5}
CPC	1.3×10^{-5}	2.0×10^{-5}
PC_4	8.2×10^{-6}	2.0×10^{-5}
CCM/DMSO	4.6×10^{-5}	6.1×10^{-5}

[a)]Concentration of the CNSs or CCM required to kill 50% of the cells in a given period.

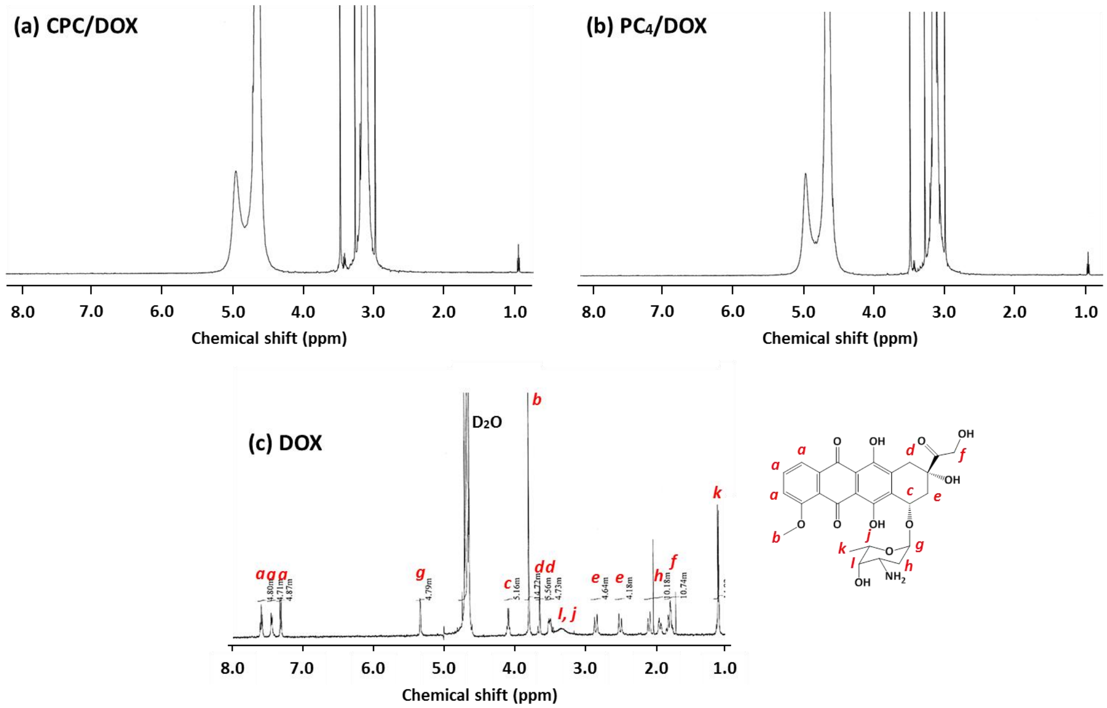

Figure 11. ^{1}H-NMR spectra of (a) CPC (100 μM)/DOX (50 μM) hybrid nanoassemblies, (b) PC_4 (100 μM)/DOX (50 μM) hybrid nanoassemblies , and (c) DOX measured in D_2O.

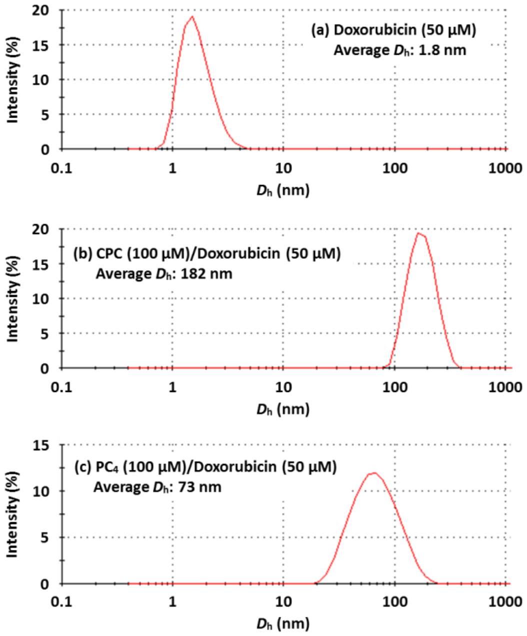

Figure 12. DLS data of (a) free doxorubicin, (b) CPC/DOX hybrid nanoassemblies, and (c) PC_4/DOX hybrid nanoassemblies measured in PBS (pH 7.4).

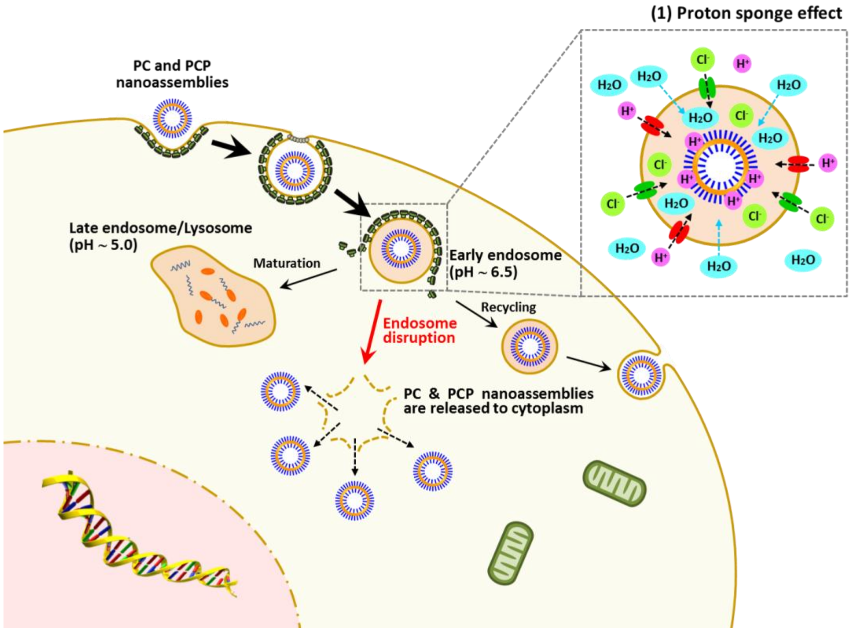

Figure 13. Schematic illustration of endosomal escape of PC and PCP nanoassemblies facilitated by curcumin segments-based proton sponge effect after cellular internalization.

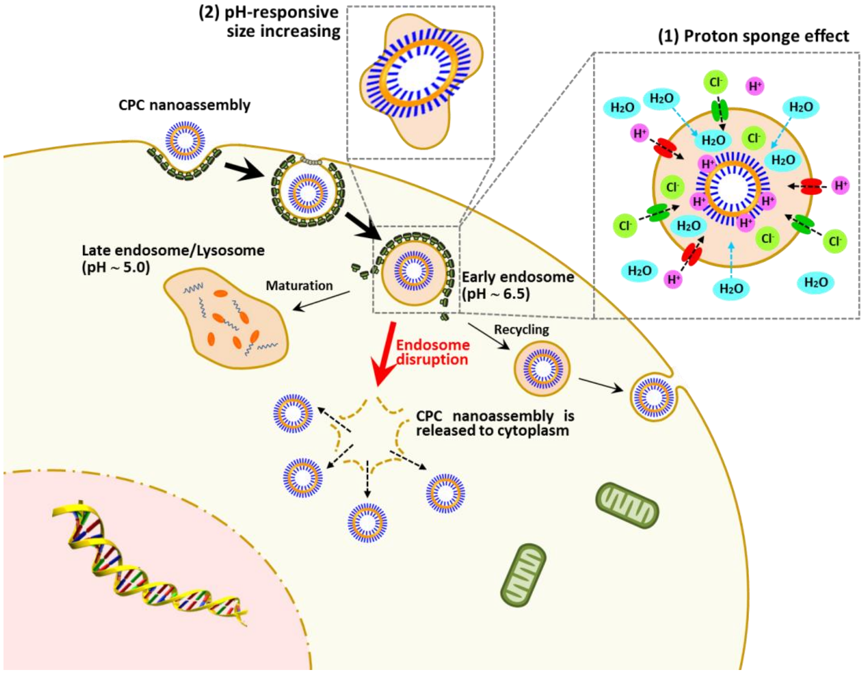

Figure 14. Schematic illustration of endosomal escape of CPC nanoassembly facilitated by curcumin segments-based (1) proton sponge effect and (2) pH-responsive size increasing effect after cellular internalization.
